# Differences in Intrusive Memory Experiences in Post-traumatic Stress Disorder after Single, Re- and Prolonged Traumatization

**DOI:** 10.3389/fpsyg.2016.00865

**Published:** 2016-06-10

**Authors:** Helge H. Müller, Sebastian Moeller, Konstanze Jenderek, Armin Stroebel, Kurt Wiendieck, Wolfgang Sperling

**Affiliations:** ^1^Department of Psychiatry and Psychotherapy, School of Medicine and Health Sciences – University Hospital – Karl-Jaspers-Klinik, Medical Campus University of Oldenburg, Bad ZwischenahnGermany; ^2^Department of Neurology, Friedrich-Alexander University Erlangen-Nürnberg, ErlangenGermany; ^3^Department of Psychiatry and Psychotherapy, Friedrich-Alexander University Erlangen-Nürnberg, ErlangenGermany; ^4^Department of Neurosurgery, Friedrich-Alexander University Erlangen-Nürnberg, ErlangenGermany

**Keywords:** post-traumatic stress disorder, intrusive memory experiences, trauma, trigger, prolonged traumatization, single traumatization, re-traumatization

## Abstract

Intrusive memory experiences (IMEs) are a common symptom of post-traumatic stress disorder (PTSD). Sensory perceptions of IMEs in the PTSD context vary substantially. The present research examined 20 patients with a single trauma, 20 re-traumatized patients and 80 Holocaust-traumatized patients who suffered from PTSD. Our results revealed significant differences in IME frequency based on the types of trauma experience. The findings suggest that patients with prolonged (Holocaust) traumata suffered from visual (65%) and combined visual/acoustic intrusive memories (29%), whereas visual memory experiences were most frequent (90%) among single-trauma patients. The trauma experience and the intrusive memory trigger stimulus were interdependent. The type of trauma critically affects the traumatic experience. Future studies should focus on these findings to improve PTSD therapeutic options.

## Introduction

According to the International Statistical Classification of Diseases and Related Health Problems, version 10 (ICD-10), and the Diagnostic and Statistical Manual of Mental Disorders 4th edition-revised (DSM IV-R), intrusive memory experiences (IMEs) are a core criterion for post-traumatic stress disorder (PTSD) diagnosis. In that context, characteristic PTSD symptoms are influenced by confrontation with trauma-associated triggers arising from all senses. Triggers must be differentiated in adverse IMEs as scenes or sensual experiences that lead to IMEs, which are defined as re-experiencing symptoms of parts of a trauma within the PTSD context. These experiences appear to be temporary, with strong inter-individual variations in the perceived intensity and extent of the re-experienced trauma (Kleim et al., 2013; Berntsen and Rubin, 2014; Muller et al., 2015).

Post-traumatic stress disorder affects up to 3% of the general population, and by definition, persists for at least 1 year (Ohayon and Shapiro, 2000; Davidson et al., 2002; Green, 2003).

The DSM defines PTSD as a hallucinogen-like, persistent perception disorder without explicitly citing the cause of the trauma (Bourne et al., 2013; Bryant et al., 2013; Nickerson et al., 2013). Recurrent memories of a traumatic experience vary among the visual, acoustic, tactile, olfactory, and gustatory perception levels and strongly differ in intensity, duration, and frequency. Improved ability to predict IME quality is thus desirable. Therefore, substantial variability exists in the traumatic event as the underlying cause of PTSD development and the trauma experience, including subjective evaluation. PTSD patients can be categorized into three groups: single-trauma patients, re-traumatized patients (single or multiple repetitions of an earlier trauma) and patients who have been traumatized over a period of years and suffered from a number of different traumatic experiences (Taubenfeld et al., 2009; Holmes et al., 2010; Bryant et al., 2011; Besser et al., 2014). Existing research provides limited knowledge regarding this heterogeneous group’s re-living of traumatic experiences through IMEs at the specific perceptual level (Hellawell and Brewin, 2004; Duke et al., 2008; Muller et al., 2015). Little is known about factors determining the quality of such experiences at the predominant perceptual level. Research on this topic has covered several areas, including examination of the underlying trauma; the command language used to represent different types of trauma; and biological aspects, such as changes in regional cerebral blood flow (Osuch et al., 2001), functional magnetic resonance imaging (fMRI) (Lanius et al., 2005) changes and changes in gender– and psychosocial-specific aspects. Several studies have shown the profound, prolonged impact of Holocaust-related traumatization (Cohen et al., 2001; Golier et al., 2006; van Rooij et al., 2014; Carmel et al., 2016). Re-traumatization is believed to cause greater PTSD symptoms than single traumatization does (Schock et al., 2010). However, few data are available for direct comparisons of these different types of trauma (Muller et al., 2015). Thus, our research objective was to compare these special traumatization groups.

The role of triggers in IMEs is also important to explore. In addition to spontaneous occurrence of IMEs, sensory stimuli known as triggers are frequently responsible for eliciting IMEs in single, re- and prolonged traumatization (Ehlers et al., 2004; Kleim et al., 2013). However, the sensory quality and threshold intensity of triggers that elicit IMEs may differ (Biermann et al., 2010; Muller et al., 2011; Post et al., 2014). Nevertheless, considering the factors influencing symptom variety might be crucial to individual therapy and to our understanding of PTSD (Holmes et al., 2010; Muller et al., 2015). We hypothesize that the frequency and quality of IMEs differ among patients with single, re- and prolonged traumatization. Although data from this study population have been published before (Muller et al., 2015), the present study reports extensive results on specific questions regarding these IME.

Therefore, the current study sought to explore the differences in trauma perceptions according to sensory modalities of IMEs among the three different groups (patients with single trauma/re-traumatization/prolonged traumatization).

## Materials and Methods

### Patients and Study Procedure

The University of Erlangen-Nürnberg Ethics-Committee approved the study. All data were obtained from personal interviews within a continuous 2-year period. All patients provided written consent to participate in the study. All patients were recruited from standardized treatment at the psychiatric ambulance at the University Hospital of Erlangen. Patients’ answers were immediately anonymized after the examination. To avoid rater bias, the interviewer was not the treating physician.

The inclusion criterion was a clinical diagnosis of PTSD by the treating physician according to DSM-IV criteria. A single rater collected all data concerning the assessment of IME- and PTSD-specific symptoms. The interviewer was blinded to the trauma type to avoid rater-related bias effects and to ensure reliability. Each inclusion interview contained a 30-min structured interview using sections of the Structured Clinical Interview for DSM-IV (including its criterion, *a traumatic event checklist*).

Patients with comorbid mental diseases other than PTSD, patients with chronic substance abuse, and patients with major medical or neurological illnesses (e.g., stroke, dementia, heart failures) were excluded from the study.

In a further evaluation of subsequent expert reports, any psychiatrically relevant shift in diagnosis or comorbidity in individuals was also recorded with respect to each specific symptom. A total of 59 patients with PTSD were initially screened for participation in the study; 19 patients had to be excluded (13 because of medical purposes and 6 because of comorbid alcohol abuse that met the DSM criteria for alcohol abuse).

The data were anonymously summarized before statistically evaluation without personal specification.

### Measures

Post-traumatic stress disorder symptom severity was assessed with the PTSD Symptom Scale-Interview (PSS-I; Foa and Tolin, 2000), which covers the 17 PTSD symptoms according to the DSM-IV (American Psychiatric Association, 2013). A validated German translation has been used (Schnyder and Moergeli, 2002). Patients were asked to identify the most traumatic event in their lives that was still bothersome. Both subjective and objective criteria were used in estimating the PTSD rates. The objective criteria were based on the following response options (1) at least twice per day; (2) more than twice per week; (3) more than twice per month; and (4) more than twice per year.

Traumatization history was categorized as single traumatization, re-traumatization (i.e., one or more repeated incidents of the primary trauma within the following 5 years) or prolonged traumatization, i.e., Holocaust traumatized patients (traumatization lasting a period of >6 months).

Intrusive memory experiences were defined as occurring when patients re-experienced parts of the original trauma within the PTSD context, as described in the DSM-IV. IMEs were assessed using the German version of the SCID PTSD (Wittchen et al., 1997). Reports of IMEs were categorized according to their frequency: (1) at least twice per day; (2) more than twice per week; (3) more than twice per month; and (4) more than twice per year. The quality and modality of IMEs were categorized as visual, acoustic, visual and acoustic or other (olfactory, gustatory, and tactile). IME triggers were defined as sensory stimuli likely to activate a known IME in an individual subject. These triggers were described independently in all three groups and categorized with respect to the stimulus modality, i.e., visual, acoustic, visual and acoustic, or other (olfactory, gustatory, and tactile).

### Characteristics of the Patient Groups

The single- and re-traumatized group consisted of 40 patients (20 patients each, with 9 females and 31 males). The mean age was 45.8 ± 11.7 years, and the age at the onset of traumatization ranged from 8 to 67 years (mean 39.10 ± 12.90 years).

The prolonged traumatization group consisted of 80 patients (47 females and 33 males) born between 1926 and 1939. The mean age was 76.2 ± 6.5 years. All 80 patients in the prolonged traumatization group were child Holocaust survivors (Muller et al., 2015).

### Statistical Analysis

For data analysis, we used a commercially available statistical program (SPSS^TM^ 19.0, SPSS Inc., Chicago, IL, USA). We tested the data for the presence of normal distribution using the Shapiro–Wilk test. All statistical tests were two sided, and a significance level of <0.05 or less was used. Differences between categorical data were tested using the *X*^2^ and Fisher exact test statistic. Given a lack of normality of data, non-parametric tests (Mann–Whitney *U* and Kruskal–Wallis *H* tests) were used for the continuous variables. We performed categorical regression by assigning numerical values to the categories, resulting in an optimal linear regression equation for the transformed variables. Linear regression analysis was performed by minimizing the sum of squared differences between a dependent variable and a weighted combination of predictor variables. The nominal categorical data were recoded into binary or contrast variables. The dependent variable was IMEs, and the predictors were the age at the time of experiencing IMEs, gender, age, and single- and re-traumatization. We controlled for age- and gender-related bias in the sample group by using Fisher’s LSD *post hoc* test.

## Results

The results revealed a highly significant difference between the levels of trauma experience that induced PTSD and the frequency of IMEs (*p* < 0.01).

In the prolonged traumatization group of Holocaust survivors, the traumatic experiences manifested for these individuals either through committing physical and emotional violence against themselves or through witnessing violence against another individual. In both the single- and re-traumatization groups, the majority (75%) of trauma experiences were a combination of personal experiences of physical and emotional violence in the form of potentially life-threatening accidents and personal physical (single-trauma group: *n* = 9; re-traumatized group: *n* = 11) or emotional (single-trauma group: *n* = 6; re-traumatized group: *n* = 4) damage. In five cases in each group, the patient was an immediate witness of serious violence to another person. The frequency distribution of IMEs in the three groups of patients with single trauma, re-trauma, and prolonged trauma is shown in **Table 1**. In the prolonged traumatization group of Holocaust survivors, the majority (59%) experienced IMEs several times per day. Having IMEs several times per week or per month was more prevalent among patients with single traumas or re-traumatization (*p* < 0.05).

**Table 1:** Trauma categories.

IME frequency	1	2	3	4
(a) Single trauma *n* = 20	2 (10%)**	8 (40%)**	10 (50%)*	0**
(b) Re-trauma *n* = 20	2 (10%)**	9 (45%)**	9 (45%)*	0**
(c) Prolonged trauma *n* = 80	47 (59%)*	26 (33%)**	7 (9%)*	0**

Frequency of IMEs in relation to the traumatization group: (1) Twice or more per day; (2) More than twice per week; (3) More than twice per month;and (4) More than twice per year. *p* < 0.05 significance level (Intergroup comparison). *Significant, **Not significant. Significant findings are indicated in bold.

The distribution of primary perceptual qualities for IMEs and triggers in the three patient groups is shown in **Table 2**.

**Table 2:** IME/trigger frequency and modality (visual, acoustic, visual and acoustic) according to primary perceptual qualities in different types of traumatization groups.

IME Frequency Modality	IME Frequency Visual	IME Frequency Acoustic	IME Frequency Visual & Acoustic	IME Frequency Other	Trigger Visual	Trigger Acoustic	Trigger Visual Acoustic	Trigger Other
Single trauma	**18**	0	2	0	**19**	0	1	0
*n* = 20	(90%)*	(0%)**	(10%)**	(0%)**	(95%)*	(0%)**	(5%)**	(0%)**
Re-trauma	10	0	**9**	1	1	0	**18**	1
*n* = 20	(50%)**	(0%)**	(45%)*	(5%)**	(5%)**	(0%)**	(90%)*	(5%)**
Prolonged trauma	**52**	5	**23**	0	**38**	5	**37**	0
*n* = 80	(65%)*	(6%)**	(29%)*	(0%)**	(48%)*	(6%)**	(46%)*	(0%)**

p < 0.05 significance level (Intergroup comparison). *Significant, **Not significant.Significant findings are indicated in bold.

## Discussion

A primary symptom of PTSD is the patient’s re-experiencing of the initial trauma through nightmares, hallucinations, emotional crises, and IMEs (Taubenfeld et al., 2009; Belleville et al., 2012; Bryant et al., 2013; Cloitre et al., 2013). This study demonstrated that the perceptive quality of IMEs is significantly associated with other clinical symptoms, such as the frequency of IMEs. The severity of traumatization was significantly associated with IMEs at several perceptual levels. Additionally, the types of stimuli that could trigger IMEs were highly dependent on the trauma experience. The most frequent triggers among Holocaust survivors with prolonged traumatization were visual and combined visual/acoustic stimuli. Purely visual trigger stimuli were most common among single-trauma patients, and combined visual/acoustic triggers were most common among re-traumatized patients. Despite efforts to establish a diagnostically uniform entity, PTSD must be considered heterogeneous in its pathogenesis and manifestation. The consequences of the aﬄicted person’s exposure to trauma differ widely according to whether the person experienced single, recurrent or prolonged exposure over a period of years (Green, 2003; Hellawell and Brewin, 2004; Duke et al., 2008).

The trauma images or IMEs at various perceptual levels extend beyond the one-dimensional vision of an ever-recurrent pictorial memory (Hellawell and Brewin, 2004; Bryant et al., 2013; Cloitre et al., 2013). IMEs that result from sensory trigger sensations do not appear to uniformly exhibit equivalent perceptual levels. Visual triggers can elicit non-visual IMEs, and acoustic stimuli can elicit visual IMEs. In the present study, this was especially evident in the re-traumatization group, in which 50% of patients had visual IMEs only, while triggers were both visual and acoustical for 90% of patients. This diversity of perceptual dominance within the context of PTSD symptoms contributes to explaining the necessary complexity of therapeutic and scientific considerations (Brady et al., 2000; Tucker et al., 2001; Keeshin and Strawn, 2014). The current study sample differs from that of previous studies that have primarily considered other specific groups of PTSD patients, such as World War II veterans, victims of rape or sexual assaults, or victims of other trauma (Green, 2003; Jones et al., 2003; Duke et al., 2008). In the group of Holocaust survivors with prolonged traumatization, IMEs most frequently occurred several times per day, whereas the single-trauma and re-traumatized patients generally experienced IMEs several times per week or month. Jones et al. (2003) noted that the perceptive quality of IMEs might be affected by the media and other technological advances, leading to increased plasticity of IME perception in various senses. Our study did not identify age- or gender-related differences in IME perception or frequency. This outcome was surprising because one could hypothesize that the influence of media may be stronger among younger PTSD patients. However, the absence of age and gender differences might be related to the small size of our study. Prolonged traumatization, with wide varieties of permanent personal or shared existential threats, results in more intensely pronounced quantitative re-experiencing of trauma than does single- or re-traumatization. This result might be expected because the incidence of PTSD is strongly related to the frequency and severity of traumatization (Howgego et al., 2005; Durai et al., 2011; Hunt et al., 2011). However, we did not assess trauma severity, which is a limitation of our study. In all three groups, visual perception predominated in the perceptual quality of IMEs. However, a combined form of acoustic and visual memories in IMEs was considerably more frequent among re-traumatized and prolonged traumatization patients than among single-trauma patients. With increasing duration, the perception of the event is represented in perceptual qualities that are more varied than in cases of single trauma, with visual perceptions being predominant. In patients with single traumatization, there was a high correlation between the perceptual quality of IMEs (in this case, visual) and the perceptual quality of the eliciting trigger as visual stimuli. In contrast, greater variance in the perceptual quality of IME triggers with combined acoustic and visual stimuli was found in patients with prolonged traumatization. In re-traumatized persons, the nearly exclusive stimulation of IMEs elicited by the combined visual and acoustic perception quality of the trigger stimuli was surprising. Another limitation of our examination was the mean age and gender heterogeneity among the three different groups; however, we controlled for this characteristic of the groups using LSD *post hoc* testing, which is suitable for random samples of that size. Holocaust survivors are elderly. Thus, the rater might have assumed that an older patient has been traumatized by the Holocaust and thus categorized them as having prolonged traumatization. Additionally, age might have affected memory functions, especially among those with prolonged traumatization. However, patients with comorbid mental illnesses, such as dementia, and patients with other medical conditions that could affect memory functions were excluded from the study. Nevertheless, comorbid mental disorders were excluded using chart diagnosis, which could lead to inclusion of patients with additional mental disorders other than PTSD. Another potential source of bias is the use of retrospective questionnaires to assess IMEs; this method may not be the most appropriate assessment method and may be subject to recall bias of unknown magnitude. We used only one rater, which could potentially lead to a systematic bias. Additionally, the role of multiple traumatic events versus long-term traumatic exposure was not completely examined using this study design. Future controlled studies should address these concerns.

The multiplicity of perception engramming and the sensory modality of perception activation increase along with the frequency and duration of trauma, as previously described (Durai et al., 2011; Hunt et al., 2011). This current examination can encourage future studies to assess how the intensity and modality of IMEs influence overall treatment and outcomes. Patients with prolonged traumatization more frequently suffer from visual and combined visual/acoustic IMEs, whereas visual IMEs occur most frequently in single-trauma patients.

Regarding events triggering IMEs, trauma experiences and the occurrence of IMEs, the elicitation of IMEs by perception-specific triggers differs among those with single, re- and prolonged traumatization. Whereas the primary visual perceptual quality of the single trauma was maintained in the trigger, the eliciting perception spectrum of triggers among patients with re- and prolonged traumatization expanded to combine visual and acoustic stimuli.

In addition to the result regarding IMEs combining both visual and acoustic types associated with increased trauma frequency and duration, the described phenomenon indicates an expansion of trigger-specific elicitation of IMEs through the combined visual and acoustic perceptual contents. Hellawell and Brewin (2004) addressed this phenomenon in relation to ordinary autobiographical memories. They found that the perceptual descriptions of IMEs were more detailed, with mentions of death, use of the present tense and fear, helplessness and horror; in contrast, ordinary memories involved secondary emotions, such as guilt or anger (Hellawell and Brewin, 2004). The trauma-dependent quality of the perception of IMEs and triggers, as described in the current population, indicates that the multiplicity of perception engramming and sensory modalities of the perception activation (trigger) increase with the frequency and duration of trauma (Durai et al., 2011; Hunt et al., 2011).

Post-traumatic stress disorder patients who were Holocaust survivors more frequently suffered from visual and combined visual/acoustic IMEs, whereas visual IMEs were most common among single-trauma patients. Regarding IME triggering events, the trauma experience and the IME trigger stimulus are related. From a neurobiological perspective, the question of whether re- and long-term prolonged traumas play different roles and have different cerebral neurogenesis correlates than single traumas remains unanswered. Future research should address these questions and consider how they may contribute to improving the understanding and treatment of different types of PTSD.

## Author Contributions

HM and WS designed the study. KJ, AS, and KW provided intellectual input and participated in the data analyses. HM and SM wrote the manuscript. WS, SM, and HM critically revised the manuscript. All authors have read and approved the final manuscript.

## Conflict of Interest Statement

The authors declare that the research was conducted in the absence of any commercial or financial relationships that could be construed as a potential conflict of interest.
